# Zinc Supplementation Prevented Type 2 Diabetes-Induced Liver Injury Mediated by the Nrf2-MT Antioxidative Pathway

**DOI:** 10.1155/2021/6662418

**Published:** 2021-07-07

**Authors:** Lechu Yu, Yuanyuan Liu, Yichun Jin, Tinghao Liu, Wenhan Wang, Xuemian Lu, Chi Zhang

**Affiliations:** ^1^Ruian Center of Chinese-American Research Institute for Diabetic Complications, The Third Affiliated Hospital of Wenzhou Medical University, Wenzhou, China; ^2^School of Pharmaceutical Science, Wenzhou Medical University, Wenzhou, China

## Abstract

Zinc is an essential trace element that is often reduced under the type 1 diabetic condition. Previous studies demonstrated that zinc deficiency enhanced type 1 diabetes-induced liver injury and that zinc supplementation significantly helped to prevent this. Due to the differences in pathogenesis between type 1 and type 2 diabetes, it is unknown whether zinc supplementation can induce a beneficial effect on type 2 diabetes-induced liver injury. This possible protective mechanism was investigated in the present study. A high-fat diet, along with a one-time dose of streptozotocin, was applied to metallothionein (MT) knockout mice, nuclear factor-erythroid 2-related factor (Nrf) 2 knockout mice, and age-matched wild-type (WT) control mice, in order to induce type 2 diabetes. This was followed by zinc treatment at 5 mg/kg body weight given every other day for 3 months. Global metabolic disorders of both glucose and lipids were unaffected by zinc supplementation. This induced preventive effects on conditions caused by type 2 diabetes like oxidative stress, apoptosis, the subsequent hepatic inflammatory response, fibrosis, hypertrophy, and hepatic dysfunction. Additionally, we also observed that type 2 diabetes reduced hepatic MT expression, while zinc supplementation induced hepatic MT expression. This is a crucial antioxidant. A mechanistic study showed that MT deficiency blocked zinc supplementation-induced hepatic protection under the condition of type 2 diabetes. This suggested that endogenous MT is involved in the hepatic protection of zinc supplementation in type 2 diabetic mice. Furthermore, zinc supplementation-induced hepatic MT increase was unobserved once Nrf2 was deficient, indicating that Nrf2 mediated the upregulation of hepatic MT in response to zinc supplementation. Results of this study indicated that zinc supplementation prevented type 2 diabetes-induced liver injury through the activation of the Nrf2-MT-mediated antioxidative pathway.

## 1. Introduction

Globally, diabetes is becoming one of the severest chronic metabolic diseases. Besides the heart and kidneys, the liver is also greatly affected by diabetes, with the pathological characteristics of hepatic dysfunction, hepatic steatosis, inflammatory cell infiltration, liver hypertrophy, and fibrotic change [1–5]. Since the liver is the main organ for glucose and lipid metabolism, diabetes-induced liver injury further enhanced the metabolic disorders of glucose and lipids [6]. Surprisingly, growing evidence has demonstrated that the mortality rate of the diabetic patients with the end-stage liver disease is higher than the mortality rate of diabetic patients with cardiovascular diseases [6, 7].

In the setting of type 2 diabetes, impaired glucose and lipid metabolism leads to glycogen deposition in hepatocytes. This damages the mitochondrial electron transport chain and induces oxidative stress in the liver [8–10]. In addition, the impaired glucose metabolism forces the liver to predominantly use lipids for energy supply. This consumes more oxygen than glucose and enhances the pressure on the electron transport chain and the subsequent oxidative stress [11, 12]. Based on this evidence, oxidative stress is considered to be key in the pathogenesis of diabetic liver injury.

Zinc is an essential trace element that plays an important role in cell growth, immunity maintenance, and gene transcription [13]. Since diabetes causes intestinal permeability to decrease and urine output to increase, a great mount of zinc is excreted from the body. This can lead to zinc deficiency in patients with both type 1 and type 2 diabetes [14–16].

Previous evidence has found that most of the beneficial effects of zinc are attributed to its antioxidative function [17–19]. Zinc deficiency enhances the imbalance of redox, which leads to deterioration and oxidative damage [20]. Previous studies have indicated that the antioxidative effect of zinc can be divided into both acute and chronic effects [21]. In regard to acute effects, zinc can directly and competitively bind to the attacking target chemical groups of peroxide and superoxide. This binding prevents these groups from being damaged [21]. Chronic zinc intake induces the expression of multiple antioxidants, especially metallothioneins (MTs). MTs are a series of proteins that are cysteine-rich and have a low molecular weight and a strong metal chelating capacity [22, 23]. The induced MTs have the capacity to scavenge oxygen free radicals and induce antioxidative effects [24]. Strong evidence demonstrates that MT is the required downstream molecule for Nrf2-mediated preventive effects of sulforaphane on diabetic cardiomyopathy [25]. Moreover, Li et al. indicated that zinc is essential for the transcription function of Nrf2 in human renal tubule cells in vitro and mouse kidney in vivo in the setting of diabetes [26].

In contrast, administration of zinc partially prevented diabetic renal damage [27]. Previous research has indicated that zinc deficiency strongly enhanced diabetes-induced liver injury likely by downregulation of Akt-GSK3*β*-Nrf2-mediated antioxidative function in type 1 diabetic mice [3]. More importantly, we also found that supplementation of zinc notably prevented the functional, structural, and biochemical abnormalities in the liver of the spontaneous type 1 diabetic mice (OVE26) associated with the upregulated hepatic MT level [4].

Evidence has demonstrated that MT alleviates cardiac dysfunction in streptozotocin-induced diabetes [28]. The role of MT in zinc-induced hepatic protection against diabetes is still unknown. At this point, there are obvious differences of the pathogenesis and symptoms between type 1 and type 2 diabetes. Type 2 diabetes is more commonly present in the clinic.

In this study, the aim is to identify whether supplementation of zinc induces beneficial effect on type 2 diabetes-induced hepatic damage. In addition, it remains to be determined whether the hepatic protective effects of zinc against type 2 diabetes are also mediated by the Nrf2-MT antioxidative pathway. In order to do that, MT-knockout (KO), Nrf2-KO, and age-matched wild-type (WT) control mice were treated with a high-fat diet (HFD) plus a single-time dose of streptozotocin (STZ) to induce type 2 diabetes. This was followed by zinc sulfate treatment for 3 months. Then, liver hypertrophy, hepatic cell death, inflammation, fibrosis, and oxidative damage were examined.

## 2. Materials and Methods

### 2.1. Ethics Statement

The experimental protocol was approved by the Committee on the Ethics of Animal Experiments of Wenzhou Medical University (SYXK2015-0009, Zhejiang, China). All surgery was performed under anesthesia induced by intraperitoneal injection of 1.2% 2,2,2-Tribromoethanol (Avertin) at a dose of 0.2 ml/10 g body weight to minimize suffering of the subjects.

### 2.2. Establishment of Type 2 Diabetic Mouse Model and Zinc Treatment

MT-KO mice (8-week-old, male, with background of 129S1/SvImJ), Nrf2-KO mice (8-week-old, male, with background of FVB), and their age-matched WT mice were obtained from Jackson Laboratory (Bar Harbor, Maine, USA). The mice were housed at 22°C with a 12 h light and dark cycle and free access to rodent chow and tap water. After a two-week acclimation, the HFD/STZ strategy was applied to the animals to induce type 2 diabetes as described previously [29].

For the first step, the mice were fed a HFD (Shanghai SLAC Laboratory Animal Co., Ltd., 40% of calories from fat) for 3 months in order to induce obesity and insulin resistance. Then, the obese mice were given a single injection intraperitoneally of STZ at 50 mg/kg body weight to induce hyperglycemia and finally establish a type 2 diabetic mouse model [30]. The age-matched nondiabetic mice were given an injection of equal volume of citrate buffer and fed a standard diet (SD, Shanghai SLAC Laboratory Animal Co., Ltd., 10% of calories from fat). Then, both diabetic and nondiabetic mice were fed by gavage with ZnSO4 solution (0.1 ml/kg body weight) for another 3 months [4]. Thereafter, all the experimental mice were sacrificed immediately. Plasma and hepatic tissues were then harvested for further analysis.

### 2.3. Measurement of Zinc Level in the Liver Tissue

The hepatic zinc level was detected by an atomic absorption spectrophotometer (Varian SpectraAA 30, Georgetown, Ontario, Canada) using air-acetylene flame after the liver tissue was digested with nitric acid [31]. By this assay, the hepatic zinc in both free and protein-bound types was measured as ng/mg wet tissue.

### 2.4. Measurement of Hepatic Function Biomarkers

Multiple biomarkers represented for hepatic function including serum plasma alanine aminotransferase (ALT), aspartate aminotransferase (AST), and alkaline phosphatase (ALP) of these mice were measured using enzymatic assay kit (Thermo Fisher Scientific, Waltham, MA, USA).

### 2.5. Histological Examination

After being fixed in 10% formalin, samples were gradient dehydrated and embedded in paraffin for 24 h. The liver tissues were cut into slices at 5 *μ*m of thickness. Then, the slices were deparaffinized using xylene and ethanol dilutions and rehydration for the further staining.

### 2.6. Terminal Deoxynucleotidyl Transferase-Mediated dUTP Nick End Labeling (TUNEL) Assay

TUNEL staining was used to determine *in situ* apoptotic cells; 5 *μ*m thick samples of tissue sections were used for the TUNEL apoptosis detection. This was done using the One Step TUNEL Apoptosis Assay Kit (Beyotime Biotech, Beijing, China) following the manufacturer's instructions. The cell level of apoptosis was detected by the Leica-TCS SP8 laser confocal microscope (200∗ amplification; Leica, Germany). Apoptotic cell death, noted in red, was quantitatively analyzed by counting TUNEL-positive cells selected randomly from 10 fields. Results were presented as TUNEL-positive cells per 10^3^ cells.

### 2.7. Detection of Hepatic Lipid Accumulation

Fresh hepatic tissue was embedded in OCT at -20°C until it became solid. The samples were then cut into cryosections with a thickness of 10 *μ*m. After this, the slices were fixed in 10% buffered formalin for 5 min at 25°C, stained with Oil Red O for 1.5 h, washed with 10% isopropanol, and then counterstained with hematoxylin (DAKO, Carpinteria, CA) for 40 s. Hepatic lipid accumulation was then detected using a Nikon microscope (Nikon, Melville, NY) at 40x magnification.

### 2.8. Western Blot

Hepatic tissues were homogenized in a lysis buffer in order to release total proteins, which were collected by centrifuging at 12,000 rpm at 4°C and for 10 min. After measurement of the protein concentration by Bradford assay, the sample of total protein was diluted in loading buffer and heated at 95°C for 5 min, then subjected to electrophoresis on an 8–10% SDS-PAGE gel. Samples were then transformed to a nitrocellulose membrane. Membranes were then rinsed in tris-buffered saline and placed in blocking buffer at room temperature for 1 h. Samples were then washed 3 times with tris-buffered saline containing 0.05% Tween 20 (TBST). The membranes were then incubated with corresponding primary antibodies overnight at 4°C. The samples were washed 3 times with TBST again, and the membranes were incubated with secondary horseradish peroxidase-conjugated antibody for 1 h at room temperature. Antigen-antibody complexes were then visualized using an ECL kit (Amersham, Piscataway, NJ, USA). The primary antibodies included those against 4-hydroxynonenal (4-HNE, 1 : 2000, Calbiochem, San Diego, CA, USA), 3-nitrotyrosine, (3-NT, 1 : 1000, Chemicon), intercellular adhesion molecule-1 (ICAM-1, 1 : 500, Santa Cruz Biotechnology, Santa Cruz, CA, USA), C/EBP homology protein (CHOP, 1 : 500, Santa Cruz Biotechnology, Santa Cruz, CA, USA), plasminogen activator inhibitor type 1 (PAI-1, 1 : 2000, BD Biosciences, Sparks, MD, USA), nuclear factor-erythroid 2-related factor 2 (Nrf2, 1 : 1000), connective tissue growth factor (CTGF, 1 : 1000), and transfer growth factor (TGF-*β*, 1 : 500). These antibodies were purchased from Abcam (Cambridge, MA, USA). Other primary antibodies, including cleaved-caspase 3 (C-cas3, 1 : 500), tumor necrosis factor-*α* (TNF-*α*, 1 : 500), cleaved-caspase 12 (1 : 1000), and Bax and Bcl-2 (1 : 1000), were purchased from Cell Signaling Technology (Danvers, MA, USA).

### 2.9. Hepatic Triglyceride (TG) Detection

Hepatic tissues were homogenized in PBS, and lipids were extracted using methanol and chloroform in a ratio of 1 : 2. The samples were dried in an evaporating centrifuge and resuspended in 1% Triton X-100. Colorimetric assessment of hepatic TG levels was performed using Thermo Fisher Scientific TG assay reagents (Thermo Fisher Scientific, MA, USA). Values were normalized to the protein concentration in homogenate before extraction, determined by the Bradford assay (Bio-Rad Laboratories, Hercules, CA, USA).

### 2.10. Statistical Analysis

Data were collected from repeated experiments and are presented as the mean ± SD (*n* = 8). One-way ANOVA was used to determine the statistical difference. If a significant difference was detected, a post hoc Turkey test was used to analyze the difference between groups. The software used was the OriginLab data analysis and graphing software (version 7.5). Statistical significance was considered as *P* < 0.05.

## 3. Results

### 3.1. The Expression Changes of Both Zinc and MT under Type 2 Diabetic Condition in Response to Zinc Supplementation

**Figure 1:**
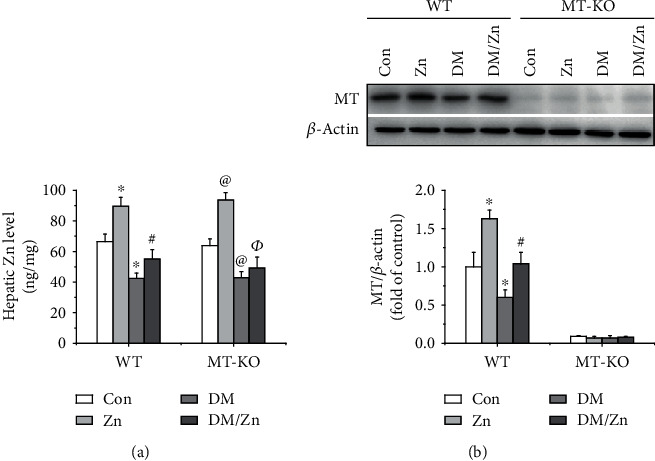
The effects of zinc supplementation on hepatic zinc and MT in both WT and WT-KO mice under type 2 diabetic condition. HFD/STZ strategy was applied in both WT and MT-KO mice to induce type 2 diabetes. Then, the diabetic and nondiabetic mice were treated with ZnSO4 for 3 months. After sacrifice, the liver tissue was isolated for hepatic zinc level measurement by an atomic absorption spectrophotometer (a). Western blot was also applied to detect hepatic MT level among groups (b). Data are presented as the mean ± standard deviation (*n* = 8/group). ^∗^*P* < 0.05 vs. the control (Con) group in WT mice; ^#^*P* < 0.05 vs. the type 2 diabetes (DM) group in WT mice; ^@^*P* < 0.05 vs. the control (Con) group in MT-KO mice; ^Ф^*P*<0.05 vs. the control (DM) group in MT-KO mice.

In the present study, we found that zinc supplementation increased the hepatic zinc level, while type 2 diabetes decreased it (Figure 1(a)) and MT level (Figure 1(b)). In MT-KO mice, the hepatic MT expression was rarely detectable in each group (Figure 1(b)). More importantly, zinc supplementation upregulated hepatic zinc and MT level under the type 2 diabetic condition (Figure 1). Additionally, the change in pattern of zinc levels among different groups was similar between WT and MT-KO mice (Figure 1(a)). This implies that the endogenous MT deficiency had no impact on hepatic zinc level.

### 3.2. The Effect of Zinc Supplement on Body Weight and Metabolic Disorders of Glucose and Lipid

**Figure 2:**
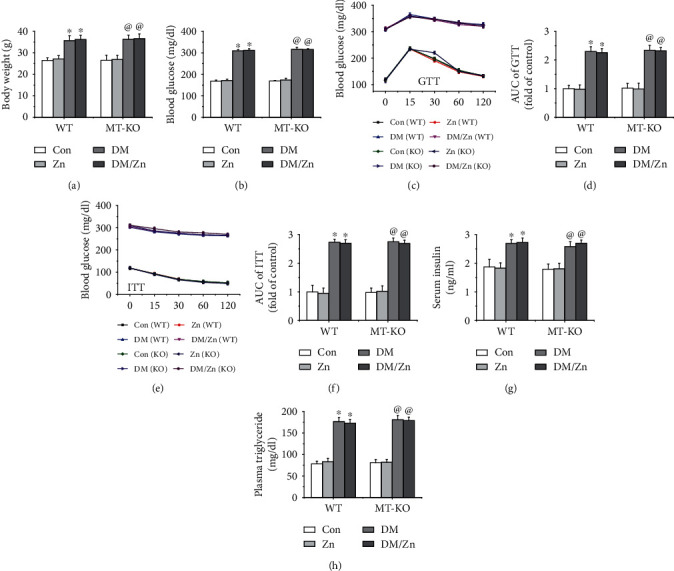
The effect of zinc supplementation on the metabolic parameters in both WT and MT-KO mice under diabetic condition. In order to identify the role of zinc supplementation on the glucose and lipid metabolic disorders, the body weight (a), blood glucose level (b), glucose tolerance (c, d), insulin sensitivity (e, f), serum insulin level (g), and plasma triglyceride level (h) were measured. Data are presented as the mean ± standard deviation (*n* = 8/group). ^∗^*P* < 0.05 vs. the control (Con) group in WT mice; ^@^*P* < 0.05 vs. the control (Con) group in MT-KO mice.

Type 2 diabetes is characterized by insulin resistance and hyperglycemia. These conditions were induced using the HFD/STZ strategy. After type 2 diabetes was established, the mice were given zinc supplementation for 3 months. Compared to the mice fed a standard fat diet, the HFD-induced type 2 diabetic mice exhibited an obvious increase in body weight (Figure 2(a)), as well as hyperglycemia (Figure 2(b)), insulin resistance characterized by decreased glucose intolerance (Figures 2(c) and 2(d)), insulin sensitivity (Figures 2(e) and 2(f)), and increased serum insulin (Figure 2(g)).

In addition, an increased plasmatic triglyceride level was also observed in type 2 diabetic mice (Figure 2(h)). After zinc supplementation for 3 months, the above biochemical abnormalities were comparable with the type 2 diabetic mice without zinc treatment (Figure 2). This indicates that the zinc supplement had no impact on the obesity or glucose and lipid metabolism in the type 2 diabetic mice. Moreover, the endogenous MT deficiency had no impact on the type 2 diabetes-induced body weight gain and hyperglycemia and hyperlipidemia with or without zinc supplementation (Figure 2).

### 3.3. Zinc Supplementation Prevented Type 2 Diabetes-Induced Hepatic Dysfunction Mediated by MT

**Figure 3:**
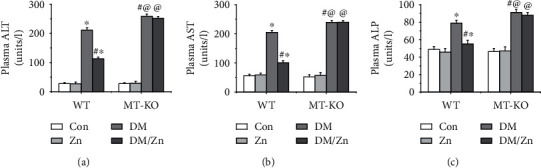
The effect of zinc supplementation on the hepatic function in both WT and MT-KO mice under type 2 diabetes. Hepatic function is regarded as the direct maker to reflect hepatic damage. The damaging markers of the hepatic function, including the levels of ALT (a), AST (b), and ALP (c), were measured by the infinity ELISA kit. Data are presented as the mean ± standard deviation (*n* = 8/group). ^∗^*P* < 0.05 vs. the control (Con) group in WT mice; ^#^*P* < 0.05 vs. the type 2 diabetes (DM) group in WT mice; ^@^*P* < 0.05 vs. the control (Con) group in MT-KO mice.

In this study, we found that 3 months after type 2 diabetes was diagnosed, obvious hepatic dysfunction was observed, characterized by the increase of plasma ALT (Figure 3(a)), AST (Figure 3(b)), and ALP (Figure 3(c)). Hepatic dysfunction in type 2 diabetic mice was strongly inhibited by zinc treatment (Figure 3), indicating that chronic zinc supplementation induced a preventive effect on type 2 diabetes-related hepatic dysfunction. In addition, we found that MT deficiency further enhanced type 2 diabetes-induced hepatic dysfunction (Figure 3). This suggested that endogenous MT played an important role in maintaining hepatic function under the type 2 diabetic condition. Additionally, zinc supplementation-induced protection of hepatic function against type 2 diabetes was blocked (Figure 3) in MT-KO mice. This suggests that MT is required for hepatic function and protection of zinc supplementation.

### 3.4. Zinc Supplementation Prevented Liver Hypertrophy in Type 2 Diabetic Mice Mediated by MT

**Figure 4:**
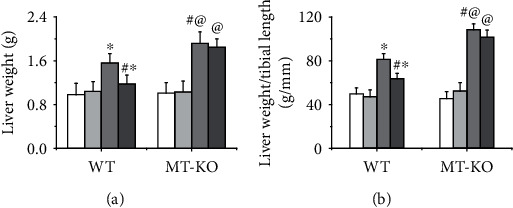
The effect of zinc supplementation on type 2 diabetes-induced liver hypertrophy in both WT and MT-KO mice. The liver weight (a), especially the ratio of liver weight to tibia length (b), is the key judgment criterion to evaluate the liver hypertrophy which was measured in the present study. Data are presented as the mean ± standard deviation (*n* = 8/group). ^∗^*P* < 0.05 vs. the control (Con) group in WT mice; ^#^*P* < 0.05 vs. the type 2 diabetes (DM) group in WT mice; ^@^*P* < 0.05 vs. the control (Con) group in MT-KO mice.

Type 2 diabetes-induced liver damage is always associated with liver hypertrophy and is characterized by the liver weight increase that was observed in the present study (Figure 4(a)). Since the body weight of mice fed with SFD and HFD was different, the ratio of body weight to tibia length was measured. We found that the ratio increased in type 2 diabetic mice and was significantly inhibited in response to zinc treatment (Figure 4(b)). This indicated that zinc supplementation induced preventive effects against liver hypertrophy in type 2 diabetic mice. Compared to the WT mice, type 2 diabetes-induced liver hypertrophy in MT-KO mice was further enhanced. This supports the idea that endogenous MT is required to keep the liver from hypertrophy (Figure 4). In addition, MT deficiency also blocked the zinc supplementation-induced preventive effect against liver hypertrophy in type 2 diabetic mice (Figure 4). This suggested that endogenous MT mediated the antihypertrophic effect of zinc treatment in the type 2 diabetic liver.

### 3.5. Zinc Supplementation Suppressed Type 2 Diabetes-Induced Inflammation and Fibrosis Depending on Endogenous MT

**Figure 5:**
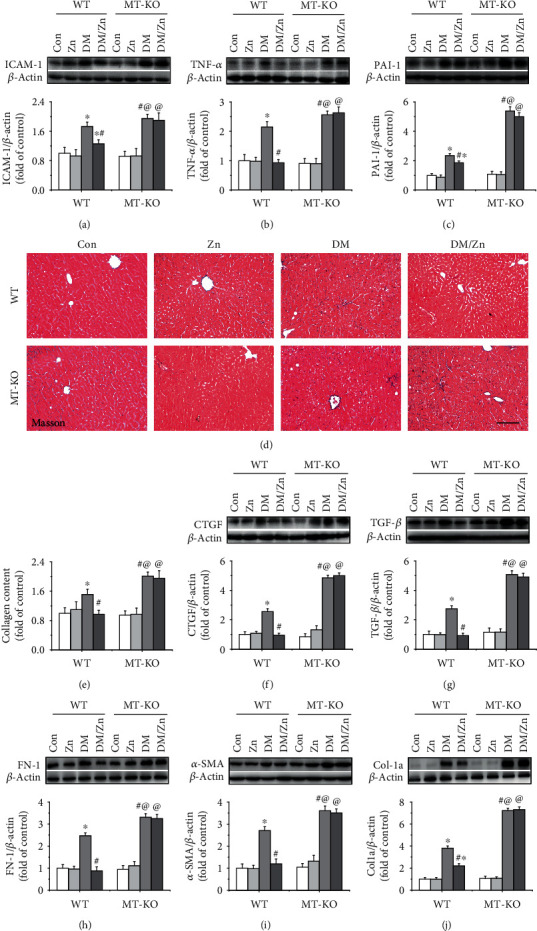
The effect of zinc supplementation on type 2 diabetes-induced inflammatory response and fibrosis. Hepatic expression of inflammatory factors, including ICAM-1 (a), TNF-*α* (b), and PAI-1 (c), was examined by western blot. Hepatic collagen accumulation was examined by Masson staining (d, e). Hepatic fibrosis was examined by western blot for the expression of CTGF (f), TGF-*β* (g), FN-1 (h), *α*-SMA (i), and Col-1a (j). Data are presented as the mean ± standard deviation (*n* = 8/group). ^∗^*P* < 0.05 vs. the control (Con) group in WT mice; ^#^*P* < 0.05 vs. the type 2 diabetes (DM) group in WT mice; ^@^*P* < 0.05 vs. the control (Con) group in MT-KO mice.

Type 2 diabetes-induced liver damage is almost always accompanied by severe hepatic inflammation characterized by the expression increases of ICAM-1 (Figure 5(a)), TNF-*α* (Figure 5(b)), and PAI-1 (Figure 5(c)).

Fibrosis is another feature of diabetes-induced liver damage that was identified by Masson staining. In this study, fibrosis was observed (blue represents fibrotic change) in both WT and MT-KO mice (Figures 5(d) and 5(e)). The diabetic-induced fibrosis was further confirmed by the upregulated expression of hepatic CTGF, TGF-*β*, fibronectin- (FN-) 1, *α*-SMA, and collagen- (Col-) 1a (Figures 5(f) and 5(j)).

Additionally, the induced inflammatory response and fibrotic effect in the WT diabetic liver were further enhanced in the MT-KO diabetic liver (Figure 5), suggesting that endogenous MT was involved in the anti-inflammatory and antifibrotic system of the diabetic liver. In WT mice, zinc supplementation showed the preventive effect on the inflammation and fibrosis under the type 2 diabetic condition (Figure 5). In this case, the beneficial effect was blocked in MT-KO mice (Figure 5). This indicated that MT is required for zinc supplement-induced anti-inflammatory and antifibrotic effect in the diabetic liver.

### 3.6. Endogenous MT Is Required for the Preventive Effect of Zinc on Type 2 Diabetes-Induced Hepatic Steatosis

**Figure 6:**
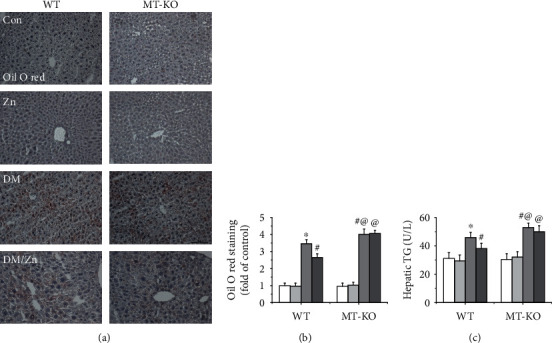
The role of MT in zinc supplementation-induced hepatic lipid-decreasing effect in type 2 diabetic mice. Hepatic lipid accumulation was examined by Oil Red O staining (positive with brown color) (a, b), and the hepatic TG level (c) was determined by TG reagent. Data are presented as the mean ± standard deviation (*n* = 8/group). ^∗^*P* < 0.05 vs. the control (Con) group in WT mice; ^#^*P* < 0.05 vs. the type 2 diabetes (DM) group in WT mice; ^@^*P* < 0.05 vs. the control (Con) group in MT-KO mice.

Hepatic steatosis is a classic feature of obesity or type 2 diabetes. In the present study, oil O red staining showed that the lipid accumulation significantly increased in liver of type 2 diabetic WT mice (Figures 6(a) and 6(b)). Similar findings were also observed for the hepatic TG level (Figure 6(c)). The phenomenon above was suppressed after treatment with zinc (Figure 6). In addition, MT deficiency further enhanced type 2 diabetes-induced steatosis and blocked zinc supplementation-induced lipid-lowering effect (Figure 6). This indicates that zinc supplementation suppressed hepatic lipid accumulation mediated by endogenous MT in type 2 diabetic mice.

### 3.7. Zinc Supplementation Induced Antiapoptotic Effect in the Type 2 Diabetic Liver Depending on MT

**Figure 7:**
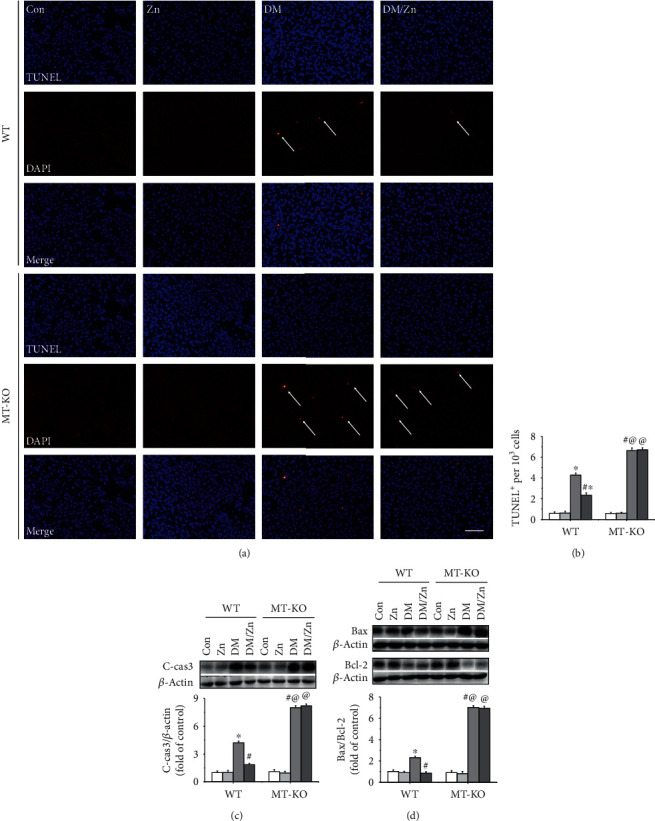
The role of endogenous MT in zinc supplementation-induced antiapoptotic effect in the liver of type 2 diabetic mice. Hepatic apoptosis was examined by TUNEL staining (a), followed by the quantitative analysis of the positive cells (b). Hepatic expression of apoptotic cell death factors, including cleaved-caspase3 (C-cas3) (c) and Bax/Bcl-2 (d), was examined by western blot analysis. Data are presented as the mean ± standard deviation (*n* = 8/group). ^∗^*P* < 0.05 vs. the control (Con) group in WT mice; ^#^*P* < 0.05 vs. the type 2 diabetes (DM) group in WT mice; ^@^*P* < 0.05 vs. the control (Con) group in MT-KO mice.

Hepatic apoptosis is regarded as the cytological basis for type 2 diabetes-induced liver damage [32]. In the present study, the hepatic apoptosis was first evaluated by TNUEL staining, which showed that the positive apoptotic liver cells, displayed in red, were hardly observed in the nondiabetic mice (Figures 7(a) and 7(b)). The positive apoptotic cells were significantly increased in the livers of type 2 diabetic mice (Figures 7(a) and 7(b)), but this was prevented by treatment with zinc (Figures 7(a) and 7(b)). These results indicate that zinc supplementation inhibited type 2 diabetes-induced hepatic apoptosis and subsequent liver injury. When compared to WT mice, the numbers of apoptotic cells were notably increased in the liver of MT-KO mice under type 2 diabetic condition (Figures 7(a) and 7(b)). This indicates MT is required for the induction of the endogenous antiapoptotic effect. Additionally, the zinc-induced antiapoptotic effect in the type 2 diabetic liver was not observed in the MT-KO mice (Figures 7(a) and 7(b)). These results suggested endogenous MT mediated the prevention of zinc treatment on type 2 diabetes-induced hepatic apoptosis. Cleaved-caspase 3, the classic apoptotic marker, was increased in the liver of WT diabetic mice and further enhanced in MT-KO diabetic mice (Figure 7(c)). Similar to the findings of TUNEL staining, MT deficiency also blocked zinc supplement-induced inhibition and increased caspase 3 cleavage (Figure 7(c)). These results confirmed that zinc prevented type 2 diabetes-induced hepatic apoptosis mediated by MT.

**Figure 8:**
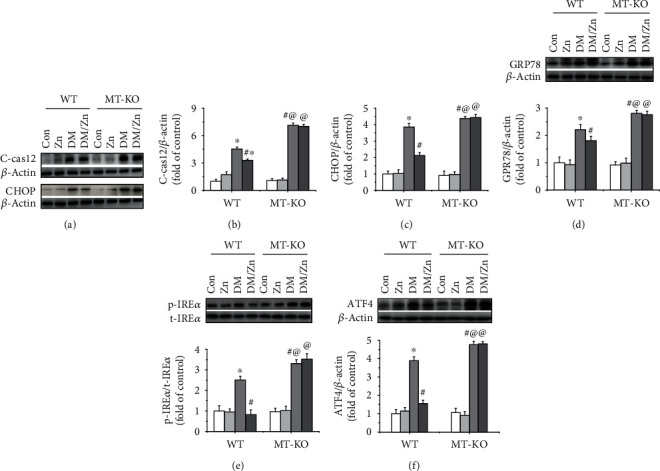
The role of endogenous MT in zinc supplementation-induced anti-ER stress in the liver of type 2 diabetic mice. Hepatic indexes of ER stress including cleaved-caspase12 (C-cas12) (a, b), CHOP (a, c), GRP78 (d), phosphorylated IRE-*α* (e), and ATF4 (f) were examined by western blot analysis. Data are presented as the mean ± standard deviation (*n* = 8/group). ^∗^*P* < 0.05 vs. the control (Con) group in WT mice; ^#^*P* < 0.05 vs. the type 2 diabetes (DM) group in WT mice; ^@^*P* < 0.05 vs. the control (Con) group in MT-KO mice.

Previous research has shown that apoptosis is mediated by the mitochondrial pathway and the endoplasmic reticulum (ER) stress pathway. These were also investigated in the present study. The ratio of Bax to Bcl-2 represented the status of the mitochondrial apoptotic pathway which was increased in the type 2 diabetic liver (Figure 7(d)). The activated ER stress pathway was also observed characterized by the expression increases of C-cas12 (Figures 8(a) and 8(b)), CHOP (Figures 8(a) and 8(c)), and GRP 78 (Figures 8(a) and 8(d)). This occurred through induction of the IRE*α*-ATF4 pathway in the liver under type 2 diabetic conditions (Figures 8(e) and 8(f)). The diabetes-activated mitochondrial and ER stress apoptotic pathways were enhanced in the diabetic liver of MT-KO mice (Figures 7(d) and 8). This indicated that MT is required for the endogenous inhibition of these two apoptotic pathways. More importantly, zinc supplementation strongly prevented the activated mitochondrial and ER stress-mediated apoptotic pathways (Figures 7(d) and 8), which was blocked in the MT-KO mice (Figures 7(d) and 8). This suggested that MT is required for zinc supplementation-induced inhibition against the activated mitochondrial and ER stress apoptotic pathway and in response to type 2 diabetes.

### 3.8. Zinc Supplementation Suppressed Type 2 Diabetes-Induced Hepatic Oxidative Stress Mediated by Endogenous MT

**Figure 9:**
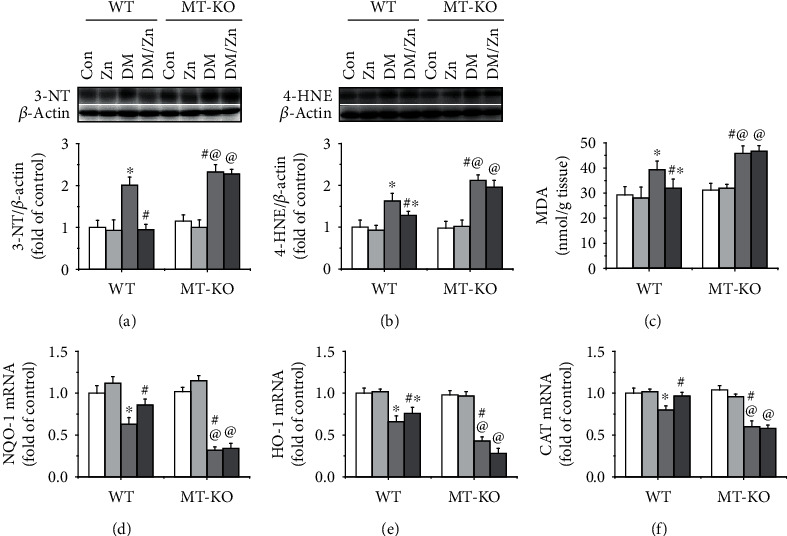
The role of endogenous MT in zinc supplementation induced an antioxidative effect in the liver of type 2 diabetic mice. Oxidative stress is regarded as the initial pathogenesis of type 2 diabetes-induced liver injury. In the present study, classic oxidative markers including 3-NT (a) and 4-HNE (b) were measured by western blot. The end-product of lipid peroxidation, MDA (c), was determined by the ELISA kit. The mRNA levels of antioxidants including NQO-1 (d), HO-1 (e), and CAT (f) were determined by real-time PCR. Data are presented as the mean ± standard deviation (*n* = 8/group). ^∗^*P* < 0.05 vs. the control (Con) group in WT mice; ^#^*P* < 0.05 vs. the type 2 diabetes (DM) group in WT mice; ^@^*P* < 0.05 vs. the control (Con) group in MT-KO mice.

Oxidative stress is regarded as the key pathogenesis of diabetic liver injury. Hence, the hepatic oxidative stress was examined by protein nitration and lipid peroxidation with western blot of 3-NT and 4-HNE, respectively, the expression of which was significantly upregulated in the liver of diabetic WT mice (Figures 9(a) and 9(b)). The hepatic content of MDA, the end-product of lipid peroxidation, was also increased in the type 2 diabetic mice (Figure 9(c)). This confirmed the severe hepatic oxidative stress of type 2 diabetes. Oxidative stress was notably suppressed after treatment of zinc (Figures 9(a)–9(c)). When compared to the WT mice, type 2 diabetes-induced hepatic oxidative stress was enhanced in MT-KO mice (Figures 9(a)–9(c)). These findings indicate that endogenous MT played an anti-oxidative role in the liver under diabetic condition. Additionally, the zinc supplementation-induced antioxidative effect against type 2 diabetes was inhibited in MT-KO mice (Figures 9(a)–9(c)). These findings suggest that endogenous MT is necessary for the antioxidative effect induced by zinc supplementation, specifically under the type 2 diabetic condition. Previous research has suggested that oxidative stress might be attributed to the impaired antioxidant transcription. We found that hepatic mRNA levels of multiple antioxidant enzymes, including *nqo-1*, *ho-1*, and *cat*, were significantly decreased under the type 2 diabetic condition (Figures 9(d)–9(f)), which were further enhanced in MT-KO mice (Figures 9(d)–9(f)). In WT diabetic mice, zinc supplementation partially prevented the reduction of antioxidant enzymes at mRNA level in the liver. This was blocked in MT-KO mice (Figures 9(d)–9(f)). These findings suggest that MT is involved in zinc supplementation-induced upregulation of multiple antioxidant enzymes at mRNA level.

### 3.9. MT-Mediated Zinc Supplementation-Induced Hepatic Protection against Type 2 Diabetes Might Be Regulated by Nrf2

**Figure 10:**
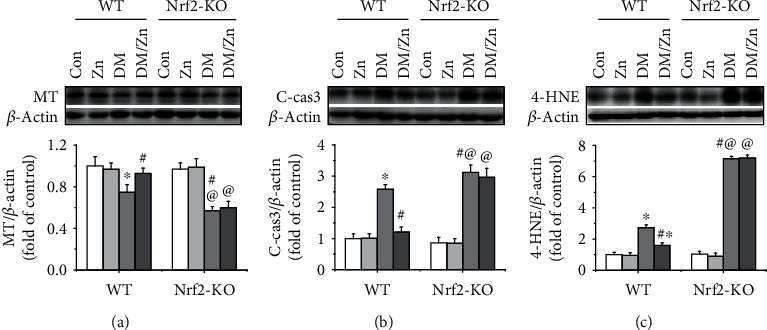
The role of endogenous Nrf2 in zinc supplementation-induced hepatic MT increase under type 2 diabetic condition. HFD/STZ strategy was applied in both WT and Nrf2-KO mice to induce type 2 diabetes, followed by 3 months of zinc supplementation. After the mice were sacrificed, the liver tissues from each group were isolated. Hepatic MT (a), cleaved-caspase3 (b), and 4-HNE (c) were determined by western blot. Data are presented as the mean ± standard deviation (*n* = 8/group). ^∗^*P* < 0.05 vs. the control (Con) group in WT mice; ^#^*P* < 0.05 vs. the type 2 diabetes (DM) group in WT mice; ^@^*P* < 0.05 vs. the control (Con) group in Nrf2-KO mice.

Since *nqo-1*, *ho-1*, and *cat* are the downstream targets of Nrf2, the relationship between Nrf2 and MT in the zinc-induced hepatic protection in Nrf2-KO mice under type 2 diabetic condition was also examined in this study. We found that type 2 diabetes decreased the hepatic MT level (Figure 10(a)). This effect was further enhanced in Nrf2-KO mice (Figure 10(a)). Supplementation of zinc significantly prevented the reduction of hepatic MT under the type 2 diabetic condition (Figure 10(a)). This result was blocked in Nrf2-KO mice (Figure 10(a)). In addition, Nrf2-KO also blocked zinc supplement-induced prevention of hepatic apoptosis and oxidative stress (Figures 10(b) and 10(c)). These results indicate that Nrf2 is required for MT-mediated hepatic protection of zinc against type 2 diabetes.

## 4. Discussion

Zinc is a trace metal and also an active component of various enzymes. Typically, the level of zinc is decreased under the type 1 diabetic condition. This is attributed to increased urination [33–35]. In addition, growing evidence has demonstrated that zinc deficiency further enhanced the type 1 diabetes-induced tissue damage of multiple organs including the heart [33, 36], kidneys [26], aorta [37], and testis [16, 38, 39]. All of the phenomenon were prevented by zinc supplementation [40–43], indicating that zinc has beneficial effects on multiple diabetic complications. Previous research has shown that the liver is another target organ of diabetes. More importantly, our previous studies indicated that type 1 diabetes-induced hepatic pathogenic damage, inflammation, oxidative stress, and insulin resistance were all exacerbated in the zinc-deficient mouse model [3]. This was notably prevented after chronic treatment with zinc [4], suggesting that zinc induces beneficial effects on the liver in type 1 diabetic mice. The current study revealed that zinc-induced hepatic protection against type 1 diabetes occurred via activation of AKT-GSK3*β*-Nrf2-mediated, antioxidative signaling [3]. Additional research by others has confirmed that administration of zinc reversed the reduction of multiple antioxidants including catalase, superoxide dismutase, glutathione-S-transferase, glutathione peroxidase, and glutathione reductase in the type 1 diabetic liver [44].

It is well known that the pathogenesis between type 1 diabetes and type 2 diabetes is different. Type 1 diabetes is regarded as absolute deficiency of insulin and is attributed to the complete damage of islet *β* cells. Type 2 diabetes occurs from relative deficiency of insulin attributed to insulin resistance. Type 2 diabetes is almost always accompanied by a lipid metabolic disorder that is characterized by the excessive lipid accumulation in the liver and severe oxidative damage [1–5].

In the setting of type 2 diabetes, the antioxidative effects of zinc supplementation were characterized by decreased hepatic MDA [45]. Another study showed that zinc supplementation in addition to strength exercise reduced the lipid accumulation under type 2 diabetic condition [46]. There has been no systematic study done to identify the role of zinc supplementation in the type 2 diabetes-induced hepatic damage that was investigated in the present study. We found that zinc supplementation notably prevented type 2 diabetes-induced liver injury including hepatic dysfunction, hypertrophy, inflammatory response, fibrosis, lipid accumulation, cell apoptosis, and oxidative stress. Next, we focused on dissecting the underling mechanism of zinc supplementation-induced hepatic protection under the type 2 diabetic condition. First, we investigated whether the above beneficial effect of zinc was attributed to the regulation of the glycemia and lipid metabolism. Previous studies demonstrated that zinc treatment did not improve, and even slightly enhanced the metabolic disorders [45, 46]. Similar results were confirmed in this study, indicating that zinc supplementation-induced liver protection against type 2 diabetes was not attributed to the maintenance of glucose and lipid metabolism. Although zinc supplementation had no effect on the hyperlipidemia, the hepatic steatosis was strongly reduced in the presence of zinc treatment in type 2 diabetic mice. Strong evidence has demonstrated that zinc can act directly on the liver and keep the liver from steatosis and apoptosis by inhibition of the eIF2*α*/ATF4/CHOP-mediated ER stress pathway [47]. These findings were also confirmed in the present study. Additionally, besides the inhibition of ER-stress pathway-related apoptosis, antimitochondrial pathway-related apoptosis was also involved in zinc supplementation-induced hepatic protection under the type 2 diabetic condition. This was characterized by the ratio decrease of hepatic Bax to Bcl-2.

The mechanism of zinc supplementation regulating the ER stress pathway or mitochondrial pathway-induced hepatic apoptosis in 2 diabetic mice remains unclear. Previous studies have demonstrated that oxidative stress can be regarded as a key pathogenesis. Kang et al. indicated that zinc treatment induced a reduction of alcoholic steatosis and apoptosis in the liver with the potential mechanism of antioxidation [48]. In the present study, antioxidative and antiapoptotic effects of zinc supplementation were also observed in the liver under type 2 diabetic condition and characterized by the reduction of 3-NT and 4-HNE levels as well as the induction of antioxidants at the mRNA level like NQO-1, HO-1, and CAT. It has been previously indicated that the induction of multiple antioxidants might contribute to the zinc supplementation-induced antioxidative effect and hepatic protection in type 2 diabetic mice. Since we did not measure ROS production, it remains to be determined if the hepatic protection of zinc also was related to the inhibition of hepatic ROS. The next issue was the mechanism of zinc supplementation-induced antioxidative effect, which finally led to the hepatic protection.

As we know, MT is a low molecular weight protein that is rich in cysteine and can be induced by zinc treatment. Kumari et al. reported that MT is a strong free radical scavenger, which was predominantly produced in the liver [49]. Later studies demonstrated the MT plays a crucial role in protecting the liver from the oxidative damage induced by a diversity of pathogenic and exposure conditions [50–52]. As the ROS scavenger, hepatic MT was induced under type 1 diabetic condition. This was regarded as a compensatory effect in response to the increased ROS. In the present study, reduced hepatic MT was observed in the type 2 diabetic mice. We assumed that steatosis and insulin resistance were also involved in type 2 diabetes and may further damage the liver. This damage might impair the MT expression.

In order to identify the role of MT in zinc supplementation-induced hepatic protection against type 2 diabetes, MT-KO mice were used. As expected, the results showed that endogenous MT deficiency further enhanced type 2 diabetes-induced hepatic damage and blocked zinc supplementation-induced hepatic protection. These results indicated that endogenous MT mediated the hepatic protection of zinc supplementation in type 2 diabetic mice.

In the present study, upregulated *nqo-1*, *ho-1*, and *cat* were observed. These are the downstream target genes of Nrf2. Strong evidence indicated that zinc is essential for the transcriptional ability of Nrf2 in tubular cells [26]. Additionally, previous research demonstrated that zinc supplementation prevented type 1 diabetes-induced hepatic damage via the activation of AKT-GSK3*β*-Nrf2 pathway [4]. Of note, Gu et al. indicated that MT is downstream of Nrf2 and partially mediates sulforaphane in the prevention of diabetic cardiomyopathy [25]. Based on the evidence above, the role of Nrf2 in zinc supplementation-induced hepatic MT increase was identified in Nrf2-KO mice. Results showed that the induced hepatic MT of zinc supplementation was blocked once Nrf2 was deficient, indicating that Nrf2 is upstream of MT and able to medicate zinc supplementation-induced hepatic protection.

In summary, we systematically identified that zinc supplementation prevented type 2 diabetes-induced hepatic damage. The mechanistic study indicated that zinc supplementation-induced hepatic protection against type 2 diabetes was due to the activation of the Nrf2-MT-mediated antioxidative pathway.

## Data Availability

All data used to support the findings of this study are included within the article.
